# Diverse Roles of Antibodies in Antibody–Drug Conjugates

**DOI:** 10.3390/ph18020180

**Published:** 2025-01-29

**Authors:** Aiko Yamaguchi, H. Charles Manning

**Affiliations:** 1 Department of Cancer Systems Imaging, The University of Texas MD Anderson Cancer Center, Houston, TX 77054, USA; ayamaguchi@mdanderson.org; 2 Cyclotron Radiochemistry Facility, The University of Texas MD Anderson Cancer Center, Houston, TX 77054, USA

**Keywords:** antibody–drug conjugates, solid tumors, antibody, cell surface antigen, ADC

## Abstract

The emergence of antibody–drug conjugates (ADCs) has transformed the treatment landscape of a variety of cancers. ADCs typically consist of three main components: monoclonal antibody, chemical linker, and cytotoxic payload. These integrated therapeutic modalities harness the benefits of each component to provide a therapeutic response that cannot be achieved by conventional chemotherapy. Antibodies play roles in determining tumor specificity through target-mediated uptake, prolonging the circulation half-life of cytotoxic payloads, and providing additional mechanisms of action inherent to the original antibody, thus significantly contributing to the overall performance of ADCs. However, ADCs have unique safety concerns, such as drug-induced adverse events related to the target-mediated uptake of the ADC in normal tissues (so-called “on-target, off-tumor toxicity”) and platform toxicity, which are partially derived from limited tumor uptake of antibodies. Identifying suitable target antigens thus impacts the clinical success of ADCs and requires careful consideration, given the multifaceted aspects of this unique treatment modality. This review briefly summarizes the representative roles that antibodies play in determining the efficacy and safety of ADCs. Key considerations for selecting suitable cell surface target antigens for ADC therapy are also highlighted.

## 1. Introduction

Antibody–drug conjugates (ADCs) are a rapidly growing class of targeted chemotherapeutic agents that are designed to mitigate the severity of side effects by selectively delivering their payloads to the tumor site. As of 2024, 12 ADCs have been approved by the U.S. Food and Drug Administration and are actively being used in the clinic, with 6 indicated for the treatment of solid tumors. Clinically approved ADCs have resulted in therapeutic responses at a level that cannot be achieved by conventional chemotherapies [1], which has catalyzed academic and industrial enthusiasm for their development.

**Figure 1:**
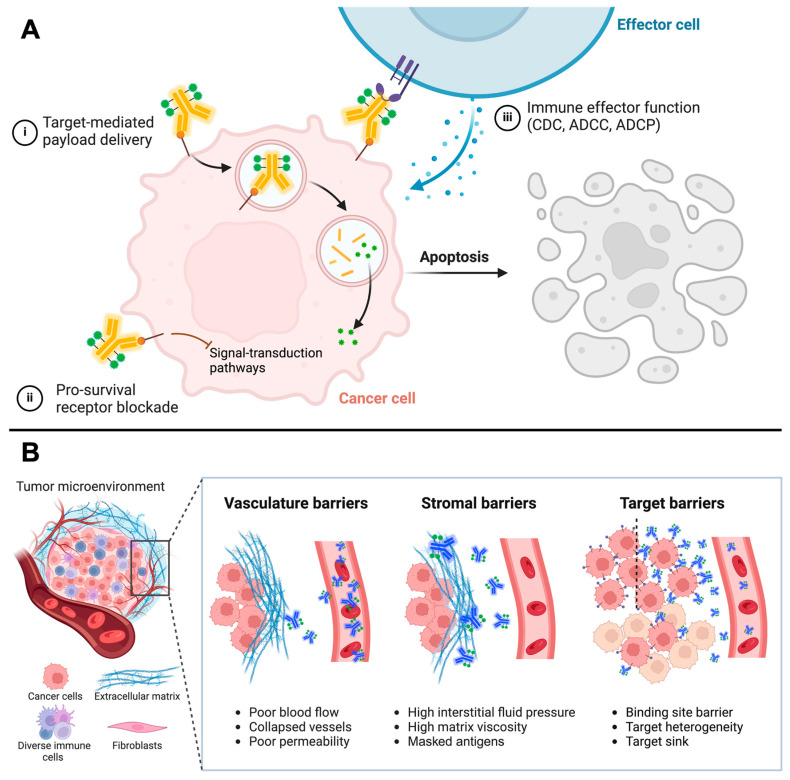
(**A**) Diverse roles antibodies play in the mechanisms of actions of antibody–drug conjugates (ADCs). (i) Specific binding and target-mediated uptake of antibodies drive the main mechanism of action of ADCs. (ii) If antibodies in ADCs retain their original activity properties, they can neutralize the antigen function or inhibit the downstream signaling pathways. iii) Interaction of antibodies with immune effector cells leads to the induction of antitumor immunity, such as complement-dependent cytotoxicity (CDC), antibody-dependent cellular cytotoxicity (ADCC), and antibody-dependent cellular phagocytosis (ADCP). (**B**) Tumor microenvironmental barriers to overcome for effective ADC therapy. Representative barriers that limit ADC uptake and distribution into solid tumors include vasculature, stromal, and target barriers. Created in BioRender.com.

Even though more than 100 ADCs have entered clinical trials, the development of new agents has been fraught with a high failure rate in the clinic, mainly stemming from their complexity. ADCs consist of monoclonal antibodies tethered to highly cytotoxic payloads, conjugated through chemical linkers. Optimization of each of these three main components, as well as the unique combination of each for a particular target, crucially impacts the therapeutic effectiveness and toxicity of ADCs [2]. Most ADCs rely on the antibodies to serve as the vehicles delivering their cytotoxic payloads selectively to the tumor site; these antimitotic agents, DNA-alkylating agents, and topoisomerase inhibitors are intracellularly released, exerting direct cytotoxic activity (Figure 1, panel A). Unlike traditional antibody-based therapy or immunotherapy agents, antibodies’ abilities to neutralize or inhibit antigen function or activate immune effector functions are not as critical when considering ADCs’ mechanisms of action. Alternatively, a key function that antibodies play in ADCs is to drive the tumor uptake of intravenously administered ADC by leveraging the specific binding of the antibody to its antigen.

The antibody–antigen targeting process is complex (Figure 1, panel B); 99% of systemically administered ADCs cannot reach the tumor due to the large in vivo distribution volume of antibodies (up to 15 L in the extracellular space in adults), specific uptake of ADCs in the actively competing non-tumor tissues that may contain the antigen, and catabolism and clearance of ADCs in the liver, kidneys, and gut [3,4]. Consequently, the systemic toxicity stemming from the exposure of healthy tissues to the cytotoxic payload remains a key issue in the development of these agents, unlike the prevailing “magic bullets” theory [1,5].

This review briefly summarizes the representative roles that antibodies play in determining the efficacy and safety of ADC treatment. Key considerations for selecting suitable cell surface target antigens for ADC therapy are also highlighted.

## 2. Antibodies’ Role in ADCs

Antibodies are expected to work as the vehicle to drive tumor uptake of intravenously administered ADCs and selectively deliver cytotoxic payloads to the tumor site via target-mediated binding following internalization. Target-mediated internalization is considered essential for ADCs carrying non-cleavable linkers; because they do not have drug release mechanisms other than antibody catabolism, they must be catabolized in lysosomes to release their active payloads [3]. Antibodies targeting internalizing antigens are preferred as well for ADCs carrying protease-cleavable linkers or acid-labile linkers, which use intracellular enzymes or acidic compartments, such as endosomes and lysosomes, respectively, for active release of the payload. However, non-internalizing ADCs can exert significant antitumor efficacy in some cases [6]. While carcinoembryonic antigen-related cell adhesion molecule 5 (CEACAM5) and B cell antigens such as CD20, CD21, and CD72 are known to internalize poorly, improved efficacy has been demonstrated with ADCs that carry cleavable linkers [7,8,9,10].

When selecting the backbone antibody for ADC construction, the antitumor activity of the antibody itself, such as direct inhibition of cellular signaling and receptor multimerization, is not considered critical, unlike in traditional unmodified antibody treatment, because the main driver of the therapeutic effectiveness of ADCs is direct cell killing by cytotoxic payloads. Nevertheless, antibodies in certain ADCs retain mechanisms of action of the unmodified original antibody, and direct antitumor activity of the antibody contributes to the overall antitumor efficacy of the ADCs. The two FDA-approved trastuzumab-based ADCs, trastuzumab emtansine and trastuzumab deruxutecan (T-DXd), are thought to retain the original mechanisms of action of their backbone antibody, trastuzumab, including inhibition of the phosphoinositide 3-kinase-AKT signaling pathway that leads to the accumulation of cyclin-dependent kinase inhibitor p27 and cell cycle arrest, inhibition of human epidermal growth factor receptor 2 (HER2) cleavage/shedding, and induction of antibody-dependent cellular cytotoxicity [11,12].

Another contributor to the antibody-mediated mechanisms of action of ADCs is the activation of immune effector functions, such as antibody-dependent cellular cytotoxicity, antibody-dependent cellular phagocytosis, and complement-dependent cytotoxicity [13]. The robustness of antibodies’ support of immune effector functions depends on their ability to engage the Fcγ receptor; these abilities differ significantly between immunoglobulin G (IgG) subclasses [14]. IgG consists of four highly conserved subclasses (IgG1, IgG2, IgG3, and IgG4), which differ in their heavy-chain amino acid sequences, how their different heavy and light chains are linked, the lengths of their hinges, and the number of disulfide bridges connecting the two heavy chains [14]. Among the four subclasses, IgG1 can most robustly engage immune effector cells, such as natural killer cells and macrophages, to elicit antibody-dependent cellular cytotoxicity and complement-dependent cytotoxicity, while IgG2 and IgG4 are insufficient or limited in their effector functions. Thus, IgG1 is the most used IgG subclass as the backbone of ADCs, while some ADCs use IgG4 or IgG2 as their backbone to manage overall toxicity when the combined toxicity of a highly potent payload and immune effector functions has become intolerable [15]. For example, gemtuzumab ozogamicin (Mylotarg^®^), inotuzumab ozogamicin (Besponsa^®^), and indatuximab ravtansine (BT062, development stage phase I/II) consist of IgG4, while two clinical-stage cMET-directed ADCs, TR1801 (development stage: phase I; NCT03859752) and SHR-A1403, use IgG2 (development stage: phase I; NCT03856541) [16,17,18].

Aside from their immune effector function, IgG subclasses differ in their reactivity to disulfide-reducing agents. A common conjugation strategy used to introduce the payload–linker module to antibodies involves cys-maleimide functionalization, which is a rapid reaction between a thiol and a maleimide that generates an iodosuccinimide product through the exposed cysteine residue of reduced disulfide bridges. Interchain disulfide bridges of IgG2s are harder to reduce than those of IgG1s, rendering them less accessible for cys-maleimide functionalization [19]. Another important consideration is the immunogenicity of antibodies. Most approved antibodies are chimeric, humanized, or human IgGs with similar constant domains, with humanized and human IgGs being the most commonly used types to construct ADCs because of the lower risk of inducing immune responses in humans than mouse or chimeric antibodies [3].

## 3. Characteristics of Promising ADC Targets in Solid Tumors

A favorable antigen expression profile is a prerequisite—albeit not sufficient—for successfully targeting a tumor with an antibody. Carter et al. described the ideal antigen expression profile for the identification of new cell surface targets for antibodies as “abundant and homogeneous antigen expression on the external surface of all tumor cells for multiple tumor types with the majority of patients for each tumor type, and absent from normal tissue” [20]. While the antibodies used in ADCs are not necessarily required to satisfy all of these criteria, as the multiple mechanisms of action can participate beyond direct binding to the antigens, meeting these criteria could still benefit the overall performance of an ADC. In the following sections, we review the roles of each factor in the context of ADCs’ performance, with emphasis on ADCs that have been clinically approved for solid tumors. Approved ADCs for the treatment of solid tumors as of December 2024 include those directed against HER2 (ado-trastuzumab emtansine [Kadcyla^®^] and trastuzumab deruxtecan [Enhertu^®^]), tumor-associated calcium signal transducer 2 (TROP2; sacituzumab govitecan [Trodelvy^®^]), Nectin 4 (enfortumab vedotin [Padcev^®^]), folate receptor mirvetuximab soravtansine [ELAHERE^®^]), and tissue factor (tisotumab vedotin-tftv [Tivdak^®^]).

Antigens’ expression on normal tissue is a key factor in their suitability for antibody targeting in oncology. Limited or preferably no antigen expression on vital normal tissue minimizes the risk of antigen-dependent toxicities in ADC treatment [20]. Most ADCs that are currently approved for hematological cancer target proteins that are highly specific to B cell lineages (CD22, CD79b, and B cell maturation antigen), with minimal to no expression in non-malignant cells. Meanwhile, in ADCs for solid tumors, the expression patterns of target proteins are not limited to malignant cells but are also expressed to some degree in non-malignant tissues. However, those target proteins are often overexpressed in tumor cells, sometimes by several orders of magnitude compared to non-malignant tissues, and the selection of the promising target is usually rationalized for these different expression profiles [21,22,23]. Antigens whose expression is restricted to non-vital organs or cell populations may still be able to be selected as targets, even if they are not differentially expressed between normal and tumor tissues [20]. However, further investigation of this hypothesis is warranted due to the recent discontinuation of upifitamab rilsodotin targeting sodium-dependent phosphate transport protein 2B (SLC34A2/NaPi2b), which is expressed in both tumor and normal tissues, such as the lungs, bronchus, and kidneys, due to the lack of efficacy at phase Ib doses, as well as on-target, off-tumor toxicities (as discussed below) observed with several clinical-stage ADCs [24,25,26].

While payload-related systemic toxicity is the most common dose-limiting toxicity of most ADC treatments, the association of ADCs with a specific target expressed in non-malignant tissues can lead to sometimes severe on-target, off-tumor toxicities. These toxicities are often manageable and reversible, with recovery possible upon discontinuation of treatment, but they often affect patients’ quality of life and necessitate attenuation of the drug-loading ratio of ADCs during their development. Trastuzumab-based ADCs (T-DM1 or T-DXd) are associated with cardiotoxicities where HER2 is physiologically expressed in the membrane of cardiomyocytes, although the incidence rate and severity are much lower than when the parent antibody itself is used as the treatment modality [27]. The anti-Nectin-4 ADC enfortumab vedotin is associated with severe skin toxicities (early-onset skin rash and hair loss) and dysgeusia [28], likely due to Nectin 4 expression in the skin and salivary glands [29]. Tissue factor-directed ADC tisotumab vedotin is associated with a substantial bleeding risk [30].

On-target, off-tumor toxicities of ADCs can become dose-limiting in the clinic when choosing targets with low-to-moderate expression levels on normal tissues, such as TROP2, Epithelial cell adhesion molecule (EpCAM), and EPH receptor A2 (EphA2). The maximum-tolerated dose may be lower than for other ADCs employing the same payload–linker combinations. For example, Dato-DXd targeting TROP2 has an attenuated drug-to-antibody ratio of 4, while other DXd-containing ADCs (e.g., T-DXd, HER3-DXd, and CDH6-DXd) have drug-to-antibody ratios of 7-8, likely due to drug-related adverse events that are consistent with TROP2 expression in normal tissues (e.g., rash, stomatitis, and mucositis) [1]. High incidences of rash and stomatitis are also observed in patients treated with other TROP2 ADCs [31,32].

## 4. Influence of Target Expression Levels on Efficacy

It has been postulated that (1) tumors have a lower limit of antigen expression that supports effective in vivo targeting and the antitumor activity of a therapeutic antibody, and (2) the density or expression level of the target antigen drives the tumor uptake and spatial distribution of therapeutic antibodies [20,33]. The same principles may apply to ADC treatment; antigen density could influence the treatment efficacy of ADCs by affecting the amount of payload delivered to the tumor tissue. However, the relationship between antigen density and efficacy is still controversial and dependent on the specific target antibody combination [34,35,36]; in addition, the limit seems to be lower than that for traditional therapeutic antibodies, as numerous other factors also influence the antitumor activity of an ADC, such as the tumor and payload type, internalization rate and efficiency, linker chemistry, and bystander effect of the conjugated chemotherapeutic payload. While 100,000 copies of CD20 were considered sufficient to effectively treat non-Hodgkin’s lymphoma with the anti-CD20 antibody rituximab, only 5000–10,000 copies of CD33 were needed to treat acute myelogenous leukemia with Mylotarg™, an anti-CD33 ADC.

The lower limit of antigen expression for targeting solid tumors is higher than that for hematological cancers. The response rates to trastuzumab as first-line treatment were 35% and 0% for patients with metastatic breast cancer with immunohistochemistry (IHC) scores of 3+ and 2+, corresponding to 2.3 × 10^6^ and 5 × 10^5^ copies of HER2, respectively [37]. Similarly, T-DM1 demonstrated an objective response rate (ORR) of 41.3% (95% confidence interval: 30.4% to 52.8%) in patients who had breast cancer with a marked overexpression of HER2, and T-DM1 showed much less antitumor activity (ORR: 20.0%, 95% confidence interval: 5.7% to 44.9%) in patients with “HER2-normal” breast cancer, defined as a HER2 fluorescence in situ hybridization ratio less than 2.0 and IHC ≤ 2+ [38].

The open-label, randomized, phase II DESTINY-Gastric01 trial (ClinicalTrials.gov identifier NCT03329690) compared the efficacy and safety of 6.4 mg/kg of DXd every 21 days with standard therapies in patients with HER2-positive gastric cancer who had received ≥2 prior treatment regimens [39]. The ORR in the DXd-treated cohort was higher among patients with an HER2 IHC score of 3+ compared with that in those with a score of 2+ and positive fluorescence in situ hybridization results (58% vs. 29%). However, in certain HER2-targeted therapeutic settings, antitumor activity has been observed for T-DXd, even when the HER2 expression level was lower than the “lower limit”. In the DESTINY-Breast06 trial, among 54 patients who had HER2-low breast cancer with an IHC score of 1+ or 2+ and a negative fluorescence in situ hybridization assay, T-DXd achieved an observed ORR of 37% [40]. In an exploratory cohort in the DESTINY-Gastric01 trial, 44 patients with HER2-low, advanced gastric cancer were treated with T-DXd and achieved an ORR of 17.5% (*n* = 7 of 40 patients) and median progression-free survival duration of 2.8 to 4.4 months, depending on HER2 expression (patients with an IHC score of 2+ survived longer) [39]. In contrast, DXd demonstrated limited activity in patients with HER2-low colorectal cancer in the DESTINY-CRC01 trial, with no objective response observed among 25 treated patients and a median progression-free survival duration of 1.4 months [41].

Sacituzumab govitecan has been approved for the treatment of patients with triple-negative breast cancer on the basis of the results of clinical trials that have been conducted with no biomarker selection, as TROP2 expression is reportedly observed in >85% of these patients [42]. However, a recent biomarker analysis from the ASCENT trial showed that sacituzumab govitecan achieved twice the ORR and progression-free survival duration in triple-negative breast cancer patients with TROP2-high or -medium as in those with TROP2-low [43].

The anti-Nectin-4 ADC enfortumab vedotin has been approved by the FDA and is administered to previously treated patients with metastatic urothelial cancer without rational biomarker-based screening. In a recent retrospective study, a multicenter cohort of patients with metastatic urothelial cancer treated with enfortumab vedotin was assessed to determine the relationship between *NECTIN4* amplification and enfortumab vedotin treatment response [44]. *NECTIN4* amplification was found to be a strong genomic biomarker to predict enfortumab vedotin response and favorable outcomes (best overall response, 96%). In contrast, the response rate in the non-amplified subgroup was 32% (*p* < 0.01), which is comparable with the expected outcomes (best overall response, approximately 40%) observed in real-world settings and the pivotal phase III EV-301 study [45,46,47]. These observations are also in line with the evidence that Nectin-4 is heterogeneously expressed in urothelial cancer molecular subtypes and is often present with reduced expression during metastatic spread [48,49]. Prospective confirmation in larger, biomarker-driven trials is awaited.

The results of these studies suggest that target expression levels often drive ADC efficacy and that there may be a lower limit for target expression levels for effective ADC treatment; this limitation varies by ADC characteristics and disease target, which is discussed further in the following sections. These observations highlight that biomarker selection should be tailored for each tumor, target, and ADC type when selecting patients for ADC treatment, even for ADCs approved in the all-comer setting.

## 5. Tumor Types and Genetic Factors

In addition to the density and expression levels, multiple other aspects of target antigens complicate drug delivery efficiency and activity in particular tumor types and impact the overall efficacy of ADCs. These include but are not limited to the spatial and temporal heterogeneity of antigen expression, the underlying genomic complexity of tumors, and differences in the tumor microenvironment [50].

As discussed above, T-DXd has demonstrated varying activity among different tumor types, with HER2-expressing breast cancer being more sensitive than gastric or colorectal cancer. One possible explanation for these varying results is the unique pattern of HER2 expression in each tumor type, as the HER2 protein often demonstrates a more heterogeneous expression pattern in gastric cancers than in breast cancers [50]. While complete membrane staining of HER2 is required for a tumor to be considered HER2-positive, an incomplete, U-shaped basolateral and/or lateral staining pattern representing gland-forming, mucin-producing carcinomas indicates HER2-positive gastric carcinoma [50].

Evidence is emerging in regard to the influence of molecular subtypes on response to ADC treatment. In the interim report of the multicenter, open-label, phase II DESTINY-Lung01 trial, the confirmed ORR of T-DXd in the *HER2* mutation cohort surpassed that in the HER2 overexpression cohort (over 60% vs. 24.5%, respectively). On the basis of these interim results, the trial was redesigned to expand on the selected group with *HER2* mutation to further explore T-DXd’s efficacy; T-DXd achieved a confirmed ORR of 55% in this cohort. A clinical-stage ADC, telisotuzumab vedotin (Teliso-V), which is composed of the anti-cMET antibody telisotuzumab conjugated to the microtubule inhibitor monomethyl auristatin E, also showed varying activity, depending on the molecular subtype of lung cancer. Teliso-V demonstrated a promising ORR in patients with previously treated c-Met-overexpressing non-squamous *EGFR*-wild-type NSCLC (36.5%; 52.2% in the c-Met-high group and 24.1% in the c-Met-intermediate group). In contrast, the ORRs were 11.6% and 11.1% in the cohorts of patients with c-Met-overexpressing non-squamous *EGFR*-mutant and c-Met-overexpressing squamous NSCLC, respectively [51]. On the basis of these results, the phase II LUMINOSITY trial (NCT03539536) was expanded on the selected group (c-Met-overexpressing non-squamous *EGFR*-wild-type NSCLC) for further evaluation of Teliso-V’s efficacy [51]. Furthermore, mechanisms of resistance to certain ADC therapies are likely to differ across different treatment settings and tumor types. Genomic and transcriptomic analyses of tumor tissue from a patient with acquired resistance to sacituzumab govitecan revealed a parallel genomic alteration in *TACSTD1/TROP2* and *TOP1*, which encode TROP2 protein and the SN-38 drug target topoisomerase 1, respectively [52].

Another important aspect of the antigen expression profile is its presence in tumors at all disease stages, including metastatic stages, broadening the opportunities for treating patients [20]. An antigen that is causally associated with disease pathogenesis would be less likely to lose targetability by antigen downregulation. While HER2 is involved in the disease pathogenesis of HER2-expressing breast carcinoma [53,54], the extent of oncogene addiction to HER2 alterations can vary on the basis of the type of genetic alteration, with oncogenes arising from gene fusions often present with the highest level of oncogene addiction, followed by other mutations [20,50].

In addition to the above-mentioned de novo mechanism of sensitivity/resistance related to the expression and molecular profiles, there are three major proposed mechanisms of acquired resistance to ADCs: antigen loss due to downregulation of antigen expression or post-transcriptional modification affecting epitope recognition; alteration of intracellular trafficking pathways; and payload resistance, largely via upregulation of ATP-binding cassette transporter proteins such as multidrug-resistant 1, multidrug resistance protein 1, and breast cancer-resistant protein [15,55]. These mechanisms are still incompletely described; a detailed understanding of the mechanisms of resistance will further facilitate the clinical utility of ADCs.

Clinical and preclinical studies evaluating rational therapy combinations to enhance ADC activity are underway to address these challenges. Examples of such combination therapy include the use of an agent that modifies vascular biology, promotes membrane expression of surface antigen, synergistically enhances payload cytotoxicity, enhances endocytosis of ADCs, or promotes recruitment and/or activation of immune effector cells [55].

## 6. Improving Conjugation and Linker Chemistry to Manage Platform Toxicity

For intravenously administrated therapeutic antibodies, multiple barriers exist to delivering the sufficient payload dose required to effectively treat solid cancers (Figure 1, panel B). These barriers include but are not limited to (1) the stromal barrier, in which diffusion across an interstitial fluid space against the pressure gradients hampers macromolecular transport [56,57], and (2) the binding-site barrier, in which antibodies are prevented from penetrating tumors by their successful binding to target antigens [58,59]. Poor vascularization and heterogeneous antigen expression in the tumor further limit the effective targeting of the tumor with ADCs. Due to the antibody’s large distribution volume in tumors in vivo, specific uptake in actively competing non-tumor tissues that may contain the antigen, and catabolism and clearance in the liver and kidneys, 99% of systemically administered ADCs cannot reach the tumor [3,4]. Consequently, while target-mediated antibody uptake drives antitumor efficacy, thus lowering the minimum effective dose, target-independent, payload-derived, off-target, off-tumor toxicities remain a key issue in the development of ADCs [1].

Colombo and Rich analyzed established maximum-tolerated doses and/or recommended phase 2 doses of approved and clinically active ADCs and found that ADCs that feature a common drug linker that is often present with similar maximum-tolerated doses, with similar toxicity profiles, independently of the target antibody; this is referred to as “platform toxicity” [1]. One factor that accounts for platform toxicity is a circulating unconjugated payload that is prematurely released from ADCs; the higher drug-to-antibody ratios and the use of cleavable linkers increase the likelihood that unconjugated cytotoxic payloads will diffuse into the circulation. T-DXd and sacituzumab govitecan were shown to exhibit a 10- to 100-fold increased dose of circulating unconjugated payload compared to T-DM1, which carries a non-cleavable linker [60,61,62]. In line with this observation, treatment with most ADCs bearing the cleavable linker system—including T-DXd, sacituzumab govitecan, enfortumab vedotin, and trastuzumab duocarmazine—is accompanied by moderate to high levels of neutropenia, alopecia, and gastrointestinal adverse events, which do not contradict with toxicities related to the released payload [63].

Besides the linker system, conjugation chemistry also partially accounts for the premature release of cytotoxic payloads in the circulation. Thiol-maleimide chemistry, which comprises many ADCs that are currently approved or in development, can undergo deconjugation of the entire drug linker from the antibody through the retro-Michael reaction [64]. Examples include T-DXd and the other deruxtecan ADCs and vedotin ADCs, including brentuximab vedotin and enfortumab vedotin. The retro-Michael reaction can reportedly induce drug-linker deconjugation from ADC at a ratio as high as 50%–75% within 7 days in plasma [1]. The deconjugated drug linker, in turn, rapidly reacts with thiol-containing plasma molecules, mainly albumin, which alone makes up 80% of the free thiols in plasma, to form new conjugates [1,65]. Colombo and Rich postulated that the albumin-binding drug-linker transfers from ADCs, potentially contributing to their antitumor efficacy. As albumin has a long half-life in humans (an average serum half-life of three weeks) through an active recycling mechanism via neonatal Fc receptor binding, long-circulating drug-linker-containing albumin could result in direct tumor uptake and undergoes gradual catabolism to release the payload. The introduction of albumin-binding moiety to small-molecule therapeutics has indeed been investigated as a strategy to enhance therapeutic efficacy by extending circulation half-life and improving tumor uptake, as small-molecule therapeutics experience short tumor retention times [66]. Albumin conjugates could also induce certain toxicities via non-specific disposition of albumin conjugates and by increasing the payload’s half-life. Albumin’s role in toxicity and/or efficacy, tied to drug-linker deconjugation, needs to be assessed; such evaluations have been difficult to conduct because the biology of albumin in preclinical models is very distinct from that in the human setting [67]. Given the striking success of deruxtecan ADCs, the potential benefit of circulating albumin–payload conjugates on the overall performance of ADCs is worth further investigation.

Research efforts to address the premature release of the payload have led to the production of highly stable, site-specifically drug-linker-modified ADCs. These homogeneous ADCs have an improved circulation half-life and higher tumor uptake, with high plasma stability, than randomly conjugated constructs; they often present with higher antitumor activity [68,69,70,71]. A recent first-in-human positron emission tomography imaging study of site-specifically radiolabeled pertuzumab further confirmed the improved tumor targetability of the site-specifically modified construct [72]. Site-specifically radiolabeled pertuzumab achieved higher tumor-to-background contrast than did randomly radiolabeled pertuzumab in the same patient. Some argue that site-specifically modified ADCs display increased on-target, off-tumor toxicity when dosed at lower doses than random coupling ADCs [15,73], but these differences cannot be solely attributed to the conjugation methods, as other factors, such as the conjugation sites and payload types, can also influence the increased occurrence of on-target, off-tumor toxicity in site-specifically modified ADCs. Further investigation in prospective, large-scale clinical trials will clarify the full potential of site-specifically modified ADCs.

## 7. Next-Generation Carriers to Manage Tumor Penetration and Systemic Toxicity

Additional efforts to address tumor penetration and systemic toxicity issues include (1) the use of next-generation carriers that are postulated to improve tumor specificity (conditionally activated antibodies and bispecific antibodies) and tumor penetration (conditionally activated antibodies and antibody fragments/nanobodies) and (2) the use of next-generation payloads, including degrader–antibody conjugates to attenuate systemic toxicity by employing targeted agents as payloads. Clinical and preclinical studies exploring the efficacy and safety of these next-generation ADCs are underway and have been comprehensively summarized in recent review articles [2,74]. This section highlights a few representative ongoing efforts related to the use of next-generation carriers.

ADCs built on bispecific antibodies (BsADCs) present a promising avenue for enhancing the therapeutic efficacy of traditional ADCs by overcoming resistance related to antigen downregulation and heterogeneity in antigen expression [75]. Certain BsADCs are expected to enhance antibody internalization and/or processing or mitigate on-target toxicity. Currently, 10 BsADCs are in clinical development, with 2 of them, JSKN003 (HER2 biparatopic) and BL-B01D1 (Zalontamab Brengitecan; EGFR×HER3), being tested in phase III clinical trials.

Biparatopic antibodies, targeting two separate epitopes of the same target antigen, can induce receptor clustering and rapid target internalization [75]. Among the 10 BsADCs being evaluated in clinical trials, six are built on a biparatopic antibody. JSKN003 is a biparatopic HER2 × HER2 BsADC conjugated to a topoisomerase I inhibitor via a dibenzocyclooctyne tetrapeptide linker on the glycan of a humanized bispecific antibody [76]. The anti-HER2 component is a recombinant humanized bispecific antibody (KN026) that targets both extracellular domains II (pertuzumab binding site) and IV (trastuzumab binding site) of HER2. Compared to T-DXd, JSKN003 demonstrated more extensive and faster internalization in HER2-overexpressing NCI-N87 cells [76]. The results from a phase I/II study of JSKN003 in patients with HER2-expressing (IHC ≥ 1+) or HER2-mutant advanced solid tumors have been recently reported [77]. JSKN003 demonstrated a promising safety profile, with no patients experiencing dose-limiting toxicity or interstitial lung disease and no treatment-emergent adverse effects leading to death or treatment discontinuation. Preliminary efficacy data were encouraging, with an ORR and disease control rate of 51.4% (95% CI: 34.4, 68.1) and 91.9% (95% CI: 78.1, 98.3), respectively. Of note, an ORR of 20.0% (95% CI: 0.5, 71.6) was observed, even in patients with HER2 IHC 1+. On the basis of this result, a phase III trial evaluating the efficacy and safety of JSKN003 in treating unresectable locally advanced or metastatic HER2-low-expressing breast cancer has recently been initiated (JSKN003-302).

BL-B01D1, an EGFR×HER3-targeting BsADC, is comprised of a bispecific antibody against EGFR/HER3 (SI-B001), a cathepsin B cleavable linker, and a topoisomerase I inhibitor (Ed-04) [78]. EGFR and HER3 are receptor tyrosine kinases belonging to the ERBB family [79], both of which are known to be strongly expressed in various solid cancers, such as lung, head, neck, esophageal, and colorectal cancers. BL-B01D1 has preliminary antitumor activity in heavily treated advanced solid tumors, with an acceptable safety profile in both phase 1a and 1b trials [80]. The preliminary efficacy and safety profiles in patients with previously treated locally advanced or metastatic urothelial carcinoma were further demonstrated in the phase 2 BL-B01D1-201 study (NCT05785039), as presented at the 2024 ESMO Congress [81]. Evaluable patients treated with 2.2 mg/kg BsADC (*n* = 27) experienced an ORR of 40.7% (95% CI, 22.4–61.2%), a confirmed ORR of 33.3% (95% CI, 16.5–54.0%), and a disease control rate of 96.3% (95% CI, 81.0–99.9%). Grade 3 or higher treatment-related adverse effects occurred in 52.9% of patients, and 35.3% experienced serious treatment-related adverse effects. No treatment-related deaths were reported.

Antibodies targeting peptide tumor antigen presented by major histocompatibility complex proteins (pMHC), known as TCR-mimic (TCRm) antibodies, offer a unique strategy. TCRm antibodies can identify tumor antigens expressed at low levels on tumor cells while being absent in normal tissues [75,82]. While ADCs built on TCRm antibodies can potentially provide antitumor efficacy with reduced on-target, off-tumor toxicity, the TCRm ADCs have thus far demonstrated suboptimal effectiveness due to the low epitope density of pMHC (tens to thousands).

Probodies are IgG molecules that are either fused with self-masking moieties at the N terminus via cleavable spacers or harbor antigen-binding sites that are designed to undergo pH-dependent conformational changes [2,83]. Various PDCs are being tested in phase I/II trials, including the AXL-targeted, pH-responsive PDC BA3011 (NCT03425279, NCT04681131); ROR2-targeted, pH-responsive PDC BA3021 (NCT03504488, NCT05271604); CD166-targeted, protease-cleavable PDC (praluzatamab ravtansine, NCT03149549 and NCT04596150); and CD71-targeted, protease-cleavable CX-2029 (NCT03543813). Although promising, challenges remain, such as identifying specific cancer subtypes that are proficient in eliciting certain conditional activation mechanisms [2].

The promise of BsADCs and PDCs has led to a global surge in interest and investment in these approaches. TCRm ADCs and ADCs targeting cancer-specific alterations, such as truncated protein (including certain mutant forms of EGFR) and post-translational modified proteins, also offer a unique opportunity to eliminate on-target, off-tumor toxicities. Nevertheless, challenges persist, primarily attributed to the complexity of solid tumors, poor penetration, limited target scope, and manufacturing complexity [75]. While these modalities are dedicated to enhancing specificity and thereby mitigating off-target toxicity and adverse effects, early clinical data [80,84,85] indicate a less promising clinical safety profile than anticipated. Relying solely on targeting mechanisms may not be sufficient to address off-target toxicity, a predominant characteristic of ADC-related toxicity.

The formation of the binding-site barrier, a virtual barrier caused by rapid binding and internalization of ADCs upon extravasation, impedes the entry of ADCs into the deeper portions of the tumor and may negatively affect the efficacy of ADCs (Figure 1, panel B). Strategies to help the ADCs and antibodies overcome the binding-site barrier include reduction in affinity, alteration in size, and coadministration of competing molecules that interfere with the binding of antibodies to the antigen [86,87,88,89]. Coadministration of high doses of unconjugated antibodies saturates receptors on the perivascular cell layers and allows for deeper tumor penetration of ADCs. This strategy is often well tolerated in the clinical setting, but the generalizability of this approach to varying tumor types and ADCs is unknown. A mathematical modeling study revealed that the benefit of co-administrating unconjugated antibody (trastuzumab) to T-DM1 was more pronounced for tumors with very high antigen expression levels, while the benefit was diminished in tumors with lower levels and payloads with the bystander effect [88]. In contrast, another computational transport analysis suggested that the increased penetration of payloads with bystander effects can partially compensate for poor antibody penetration, but larger antibody doses still result in further improvement in penetration and efficacy [90]. A preclinical study suggested the possible influence of dissociation rate constant on the therapeutic efficacy of tissue factor-directed ADCs [91]. Although theoretically reasonable, the observed therapeutic benefit was subtle. Further experimental validation investigating generalizability and optimum range of dissociation rate constant for different types of targets is warranted to prove this hypothesis.

The rate of passive diffusion of a molecule in tissue is inversely correlated with its molecular size. Consequently, antibody fragments and nanobodies (variable domain of heavy-chain-only antibody; VHHs, 12–17 kDa) exhibit faster vascular permeability and better tissue penetration than intact IgG molecules, resulting in more homogeneous distribution in solid tumors [89]. VHH drug conjugates (nADCs) can potentially compete with traditional ADCs due to their distinct pharmacological benefits, including superior solid tumor penetration, enhanced stability, relatively low production cost, ease of modification by genetic engineering means, format flexibility, low immunogenicity, and modularity. Furthermore, VHHs can access epitopes that conventional antibodies cannot, such as clefts on a protein’s surface, by leveraging long CDR3 loops [92]. The main disadvantages of nADCs include their rapid renal clearance and kidney retention. While advantageous in mitigating off-target toxicities derived from the uptake of intact ADCs into normal cells and premature payload release, the fast clearance may compromise therapeutic efficacy that requires a more prolonged presence in the bloodstream. The kidney retention of VHHs, which is often significantly influenced by polar residues in the C-terminal amino acid tag (e.g., poly-Histidine tags), may lead to kidney impairment by concentrating the carried payload in the glomerulus [89,93]. The short half-life can be addressed with additional formulations, including PEGylation, fusion to serum proteins or an IgG Fc-domain, multimerization, or the introduction of a serum-albumin-binding domain [90]. However, their effectiveness in enhancing the efficacy or safety of nADCs remains inconclusive. Addressing these challenges could potentially improve the pharmacological and safety profiles of nADCs and facilitate the clinical translation and implementation of this promising drug format.

## 8. Summary and Concluding Remarks

Antibodies play critical roles in ADC efficacy by providing tumor specificity through target-mediated uptake, prolonging the circulation half-life of cytotoxins, and providing additional mechanisms of action, such as receptor neutralization, downstream signaling pathway inhibition, and immune effector function activation. Next-generation ADCs are expected to overcome on-target, off-tumor toxicity and limited tumor penetration, partially accounting for platform toxicity. Target diversification is another area of intensive research that aims to broaden the patient population that might benefit from this innovative treatment modality.

While the identification of antibody targets has traditionally relied on differential expression of messenger RNA between tumor and healthy tissues, gene expression alone is not the sole determinant of target expression due to post-transcriptional and -translational changes [94]. As an alternative, identifying cell surface antigens through proteomic approaches can yield targets with functional importance and translational potential [95]. The cancer surfaceome approach, for example, provides an unbiased, functional target discovery platform to query changes in the tumor compared to in healthy tissues, thus enabling the discovery of more tumor-specific antigens that are suitable for specific tumor subtypes or of new targets that have undergone tumor-specific post-translational modification. Efforts have been made to diversify targets with potentially higher tumor specificity and to manage toxicity while maintaining the antitumor efficacy and improved clinical translation of ADCs.

To uncover therapeutic surface antigens for precise ADC targeting, Fang et al. compiled comprehensive transcriptomic, proteomic, and genomic data encompassing 19 types of solid cancer, as well as normal tissues [73]. They tested currently available drugs for treating solid cancers (i.e., HER2, Nectin-4, and TROP2) and found that target antigens had limited expression in normal tissues and significantly different expression profiles. Additionally, the performance of ERBB2 on breast invasive carcinoma and bladder urothelial carcinoma was found to be most prominent among all target-indication combinations, although it was moderately expressed in many normal tissues.

While predicting clinical dosing from preclinical rodent models is difficult, improvements in prediction can aid in determining the translational potential of ADCs in the development stage. Rubahamya et al. devised a prediction model to estimate working clinical doses from preclinical rodent models [96] and showed that currently approved ADCs for the treatment of solid tumors had substantial efficacy in some mouse models when administered at a similar weight-based (mg/kg) dose to that in humans. Mechanistically, equivalent dosing results in a similar drug concentration and tissue penetration in the tumor due to the unique delivery features of ADCs. Combined with computational approaches, these scaling concepts may aid in the evaluation of new agents and the design of maximally effective therapeutics [90].

The emergence and development of ADCs in the past few decades has been an important step forward in oncology: ADCs have transformed the treatment of a variety of cancers by providing efficacy at a level that cannot be achieved with conventional chemotherapeutic agents. Antibodies drive the tumor uptake of co-delivered cytotoxic payloads and offer a unique way to improve the tumor specificity of this innovative treatment modality. Unique combinations of antibody, linker, and payload influence the overall performance of ADCs; thus, careful consideration from a multifaceted perspective is required when selecting suitable cell surface target antigens. Target diversification and carrier innovation are rapidly progressing, along with improvements in other important components of ADCs; the exponential growth of the ADC landscape holds promise to eradicate difficult-to-treat solid tumors.

## Data Availability

Data sharing is not applicable.
